# Metachronous Pineal Germinoma 11 Years After Total Resection of a Mature Teratoma

**DOI:** 10.1111/neup.70073

**Published:** 2026-08-07

**Authors:** Shunsuke Koga, Ilya M. Nasrallah, Rachel Blue, Juan Pablo Ospina, Steven Brem, Jason Shpilsky, MacLean Nasrallah, Zissimos Mourelatos

**Affiliations:** ^1^ Department of Pathology and Laboratory Medicine Hospital of the University of Pennsylvania Philadelphia Pennsylvania USA; ^2^ Department of Radiology Hospital of the University of Pennsylvania Philadelphia Pennsylvania USA; ^3^ Department of Neurosurgery Hospital of the University of Pennsylvania Philadelphia Pennsylvania USA; ^4^ Department of Neurology Hospital of the University of Pennsylvania Philadelphia Pennsylvania USA; ^5^ Division of Hematology Oncology, Department of Medicine Hospital of the University of Pennsylvania Philadelphia Pennsylvania USA

**Keywords:** follow‐up studies, germinoma, neoplasms, pineal gland, second primary, teratoma

## Abstract

Central nervous system germ cell tumors are uncommon and include germinomas, teratomas, and other nongerminomatous germ cell tumors. Mature teratomas are usually cured by complete resection, but rare patients later develop a histologically distinct malignant germ cell tumor. We report a man who presented at age 20 years with a cystic pineal lesion. Gross‐total resection at age 21 years showed a mature teratoma with bronchogenic differentiation and no overt immature or malignant component on H&E. Serial postoperative MRI for 3 years showed no recurrence. Eleven years after surgery, he developed headache, gait disturbance, and confusion due to a 4.6‐cm pineal mass with obstructive hydrocephalus. Serum alpha‐fetoprotein was normal, whereas beta‐human chorionic gonadotropin (β‐hCG) was elevated. The resected tumor showed sheets of large round cells with clear cytoplasm and dense lymphocytes. Immunohistochemistry demonstrated strong nuclear OCT4 and SALL4 and membranous c‐KIT, with focal β‐hCG‐positive syncytiotrophoblastic giant cells. No nongerminomatous germ cell tumor component was identified, supporting a diagnosis of germinoma with focal syncytiotrophoblastic giant cells. After this diagnosis, retrospective evaluation of the initial tumor showed multifocal, patchy OCT4 immunoreactivity in scattered cells along the ciliated respiratory‐type epithelial lining. SALL4, c‐KIT, and PLAP were focally positive in corresponding regions. These findings raised the possibility of an occult germ cell marker‐positive population, although no discrete expansile germinoma component was identified. This case illustrates that metachronous germinoma can develop after a long disease‐free interval following resection of a mature teratoma and highlights the value of retrospective pathologic evaluation and long‐term surveillance.

## 1 Introduction

Central nervous system (CNS) germ cell tumors (GCTs) are rare neoplasms that account for approximately 3% of primary brain tumors in children and adolescents [1]. These tumors arise predominantly in midline structures, most often the pineal and suprasellar regions, consistent with the biology of primordial germ cell migration [1]. In the 2021 World Health Organization classification of CNS tumors, CNS GCTs include mature teratoma, immature teratoma, teratoma with somatic‐type malignancy, germinoma, embryonal carcinoma, yolk sac tumor, choriocarcinoma, and mixed GCT [2]. In clinical practice, these tumors are often separated into germinoma, teratoma, and other nongerminomatous GCTs (NGGCTs) because prognosis and management differ among these groups [3].

Mature teratomas are usually curable with complete resection [4], whereas germinomas are malignant but highly radiosensitive and chemosensitive, and combined‐modality therapy achieves excellent long‐term survival [5]. In contrast, NGGCTs such as yolk sac tumor, choriocarcinoma, and embryonal carcinoma have poorer outcomes and require multimodal therapy [3, 6]. Despite the generally favorable outcome for resected mature teratomas, rare cases have been documented in which a second, histologically distinct malignant GCT develops years to decades later within the CNS. This phenomenon, termed metachronous intracranial GCT, is extremely rare but clinically important because it may occur after long disease‐free intervals and in the absence of residual tumor on imaging [7, 8, 9, 10, 11].

Here, we report an adult patient who developed pineal germinoma with focal syncytiotrophoblastic giant cells 11 years after gross‐total resection of a pineal mature teratoma. The initial tumor showed no overt immature or malignant component on original H&E examination. Retrospective review after diagnosis of the metachronous germinoma identified rare atypical cells within or adjacent to the ciliated respiratory‐type epithelium. OCT4 showed multifocal, patchy immunoreactivity in scattered cells along the epithelial lining, with focal SALL4, c‐KIT, and PLAP immunoreactivity in corresponding regions. No discrete germinoma component was identified. The long interval, same‐site occurrence, and change in histology support classification as a metachronous intracranial GCT. This case highlights the need for meticulous pathologic evaluation of the index teratoma and prolonged surveillance.

## 2 Clinical Summary

**FIGURE 1:**
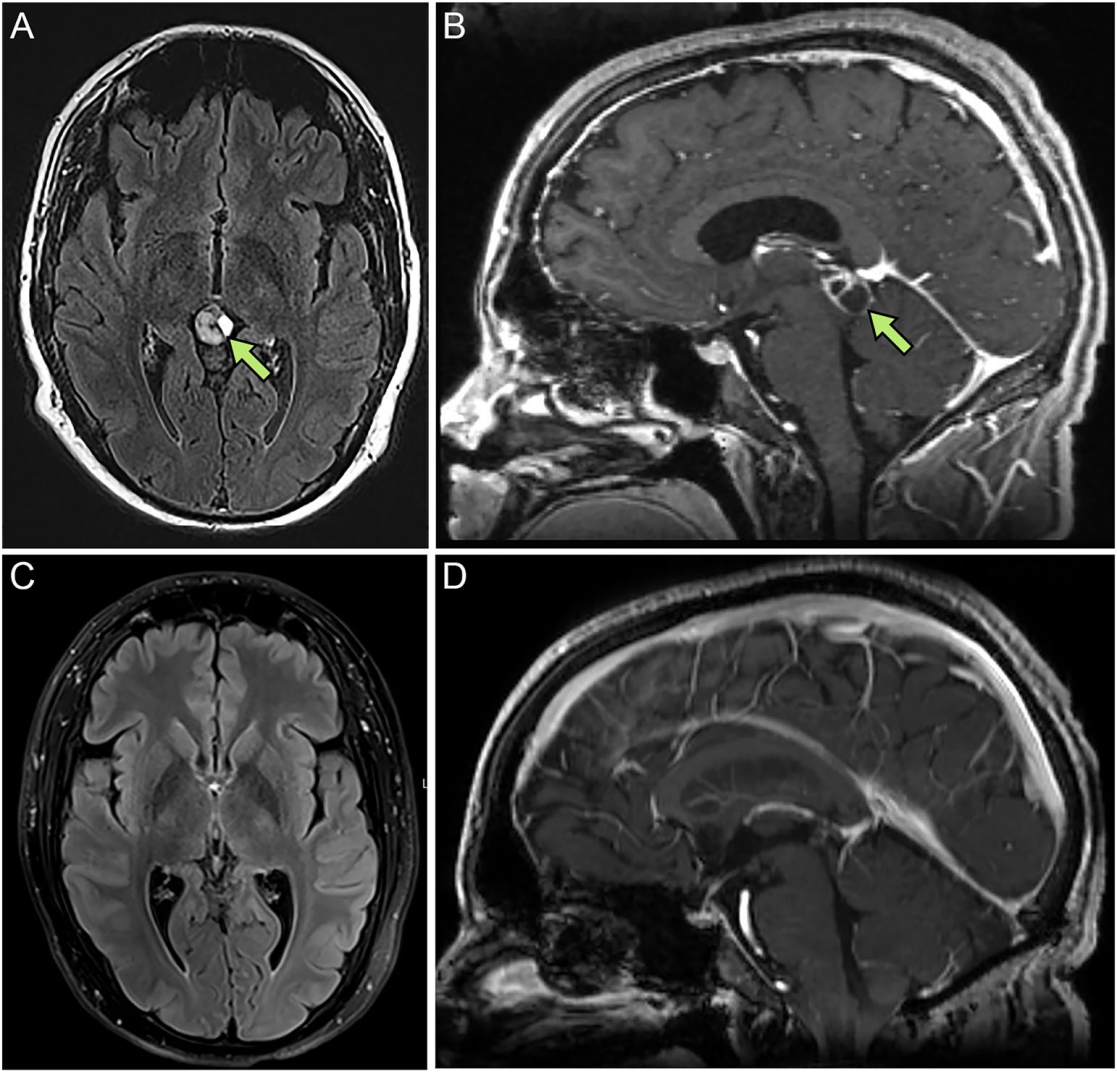
MRI of the initial pineal lesion and surveillance imaging after resection. (A) Axial T2‐weighted FLAIR image shows a lobulated, multicystic mass (arrow) centered in the pineal region without invasion of adjacent brain. (B) Sagittal postcontrast T1‐weighted image shows only thin peripheral enhancement without a solid or nodular enhancing component (arrow); the lesion has mild mass effect on the tectum with mild narrowing of the cerebral aqueduct but no hydrocephalus. Surveillance axial T2‐weighted FLAIR (C) and sagittal T1 post‐contrast (D) images at age 24 years (approximately 3 years after resection) show no evidence of recurrence.

A previously healthy man presented at age 20 years with intermittent headaches for more than 1 year and a subjective change in visual acuity. Brain MRI at age 21 identified a lesion in the pineal gland with a multiloculated appearance. He remained neurologically intact without hydrocephalus, and observation was initially chosen. A follow‐up MRI 11 months later showed a 1.8 × 1.6 × 1.6 cm lobulated, multicystic pineal mass with thin peripheral enhancement (Figure 1A,B) but no abnormal diffusion restriction or hemorrhage. The lesion abutted the cerebral aqueduct with mild mass effect on the tectum, but without evidence of hydrocephalus. At that time, the radiologic impression favored a pineocytoma or a complex pineal cyst. He underwent suboccipital craniotomy at age 21, and postoperative MRI demonstrated gross‐total resection (not shown). Histopathologic examination confirmed a mature teratoma. During postoperative follow‐up, serum AFP was 2 IU/mL approximately 4 weeks after surgery and 1 IU/mL approximately 6 and 10 months after surgery; serum β‐hCG was 5 mIU/mL at the same postoperative time points (all within the reference ranges). No adjuvant chemotherapy or radiotherapy was administered after the initial resection. Continued annual MRI surveillance had been recommended, and MRIs obtained annually through age 24 years showed no recurrence (Figure 1C,D). After this surveillance period, the patient received care outside our health system, and no further surveillance MRI was obtained during that time.

**FIGURE 2:**
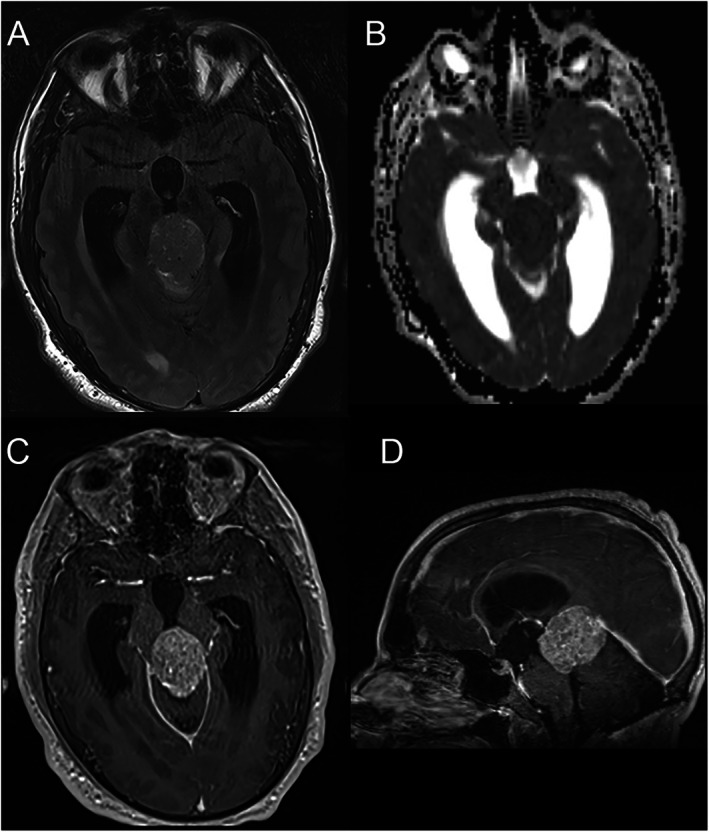
MRI at presentation of the metachronous pineal germinoma. (A) Axial T2‐weighted FLAIR image shows a lobulated extra‐axial, solid mass centered in the pineal region, moderate obstructive hydrocephalus, enlargement of the third and lateral ventricles, and periventricular FLAIR hyperintensity indicating transependymal CSF flow. (B) Axial apparent diffusion coefficient map shows low signal in the mass, consistent with diffusion restriction. (C and D) Axial and sagittal post‐contrast T1‐weighted images show heterogeneous enhancement and mass effect on the tectum and midbrain.

Eleven years after the index resection, at age 32 years, he presented with several weeks of dizziness, right‐sided headaches, gait instability, and confusion. MRI showed a 4.6‐cm heterogeneously enhancing extra‐axial solid mass in the pineal region with moderate obstructive hydrocephalus (Figure 2A–D). Symptoms improved after external ventricular drain placement. Serum alpha‐fetoprotein (AFP) was within normal limits, whereas beta‐human chorionic gonadotropin (β‐hCG) was elevated at 58.2 IU/L. Given concern for recurrent teratoma, repeat resection was performed. Intraoperative frozen section of a scant specimen with extensive crush artifact showed lesional cellular tissue, and the final diagnosis was deferred to permanent sections. At reoperation, dense postoperative scarring was encountered along the prior supracerebellar infratentorial corridor. The tumor appeared fibrous and was tightly adherent to the deep venous complex near the vein of Galen. Because the most scarred superior tumor–venous interface could not be safely separated, maximal safe resection was performed, leaving adherent tumor in place to avoid injury to the deep venous system and brainstem. Histopathologic examination confirmed germinoma with focal syncytiotrophoblastic giant cells.

Postoperatively, MRI confirmed subtotal resection, and a ventriculoperitoneal shunt was placed 2 weeks later for persistent hydrocephalus. His gait instability and confusion resolved, and his functional status returned to baseline. Staging MRI of the entire spine showed no evidence of leptomeningeal disease, and cerebrospinal fluid cytology was negative. He subsequently received four cycles of carboplatin and etoposide, which were completed approximately 4 months after surgery. This was followed by whole‐ventricular proton radiotherapy with a boost, delivered as 24 Gy in 16 fractions followed by 12 Gy in 8 fractions, for a total dose of 36 Gy. Radiotherapy was completed approximately 7 months after surgery. At the most recent follow‐up, approximately 8 months after surgery and 6 weeks after completion of radiotherapy, he had returned to work and reported no new or worsening neurologic symptoms. Brain MRI showed a slight interval decrease in solid nodular enhancement in the pineal region, from 1.2 × 0.8 × 0.7 cm to 0.9 × 0.5 × 0.5 cm, without new or progressive lesions. Continued radiographic surveillance was planned.

## 3 Pathological Findings

**FIGURE 3:**
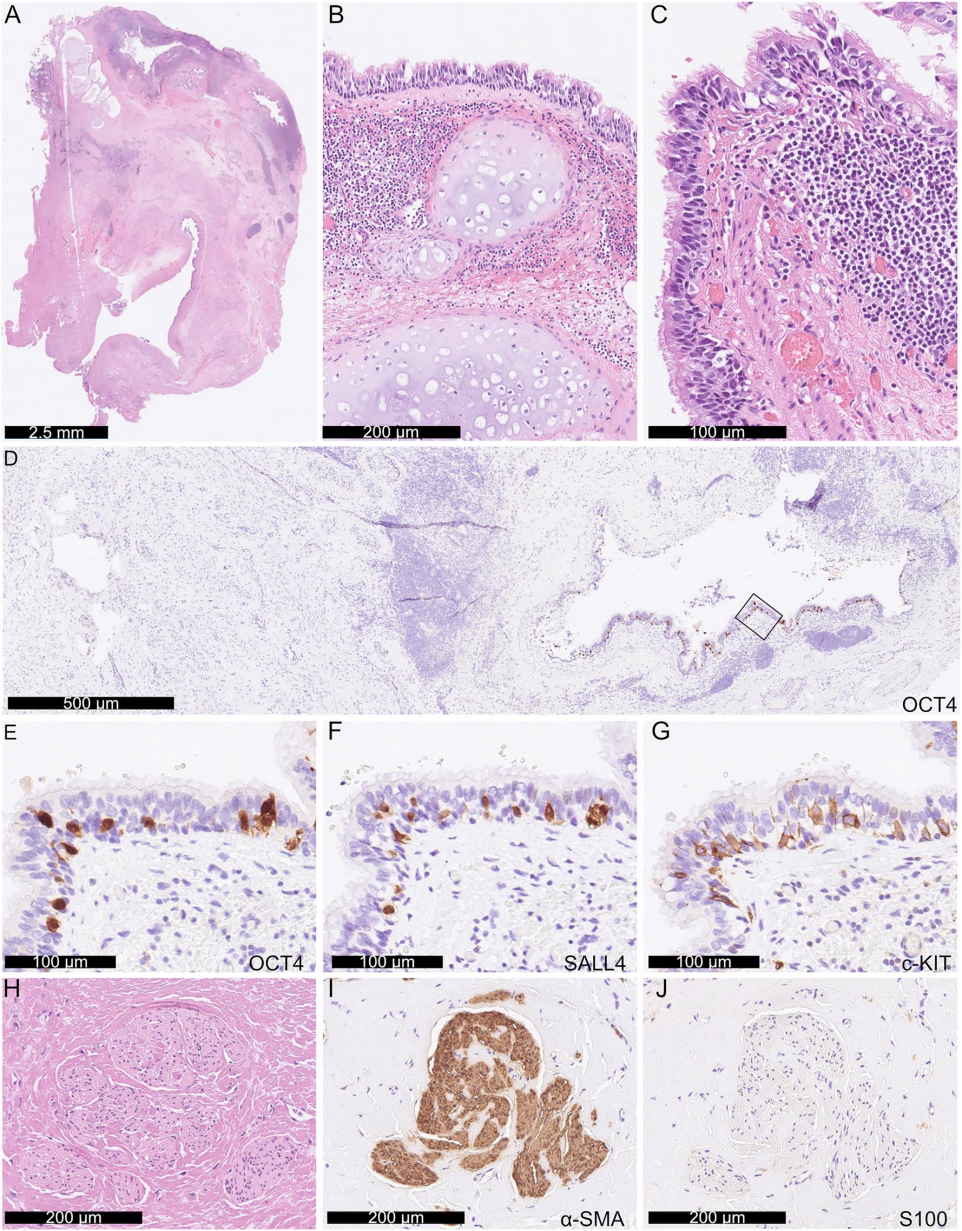
Histopathology and retrospective immunohistochemical evaluation of the initial pineal mature teratoma. (A) Low‐power H&E shows a multiloculated cystic lesion with a thick fibrous wall. (B) H&E shows ciliated respiratory‐type epithelium adjacent to mature hyaline cartilage, supporting bronchogenic differentiation. (C) Higher‐magnification H&E shows rare atypical cells within or adjacent to the ciliated respiratory‐type epithelium, with adjacent lymphoid aggregates. (D) Low‐power OCT4 immunohistochemistry demonstrates multifocal, patchy nuclear staining in scattered cells along several discontinuous segments of the respiratory‐type epithelial lining. Adjacent lymphoid aggregates and stromal components show no convincing immunoreactivity. The boxed region is shown at higher magnification in panel E. (E) Higher‐magnification OCT4 immunohistochemistry shows nuclear staining in scattered cells within the epithelial lining. (F) SALL4 and (G) c‐KIT show focal immunoreactivity in the corresponding epithelial region in adjacent sections. (H) H&E shows mature spindle‐cell tissue in the cyst wall. (I) α‐SMA confirms smooth muscle differentiation. (J) S100 is negative in the spindle‐cell tissue. Scale bars are indicated in each panel.

Pathologic examination of the initial pineal lesion showed a mature teratoma with bronchogenic differentiation (Figure 3A). The cyst wall was lined by ciliated respiratory‐type epithelium with apical cilia. Mature hyaline cartilage was present adjacent to respiratory‐type epithelium and lymphoid aggregates, supporting bronchogenic differentiation (Figure 3B). Mucous glands were also present. No immature or overt malignant GCT component was identified on the original H&E sections. Immunohistochemistry for germ cell markers, including OCT4, SALL4, c‐KIT, and PLAP, was not performed at the time of the original diagnosis.

After diagnosis of the metachronous germinoma, the archival tissue from the initial tumor was retrospectively reviewed. Rare atypical cells were identified within or adjacent to the ciliated respiratory‐type epithelium (Figure 3C). Retrospective immunohistochemistry for OCT4 demonstrated multifocal, patchy nuclear immunoreactivity in scattered cells along several segments of the ciliated respiratory‐type epithelial lining (Figure 3D). A representative area is shown at higher magnification in Figure 3E. SALL4 and c‐KIT showed focal immunoreactivity in the corresponding epithelial region in adjacent sections (Figure 3F,G), and PLAP was also focally positive (not shown). No convincing immunoreactivity was identified in hyaline cartilage, lymphoid aggregates, or the other mature tissue components in the examined sections. The immunoreactive cells did not form confluent clusters or a discrete expansile population with classic germinoma morphology. These findings raise the possibility of an occult germ cell marker‐positive cell population in the initial tumor but are insufficient for a definitive diagnosis of mixed GCT.

The lesion also contained mature mesenchymal elements, including smooth muscle and adipose tissue. H&E showed mature spindle‐cell tissue in the cyst wall (Figure 3H). α‐SMA confirmed smooth muscle differentiation (Figure 3I), whereas S100 showed no immunoreactivity in the spindle‐cell tissue (Figure 3J).

**FIGURE 4:**
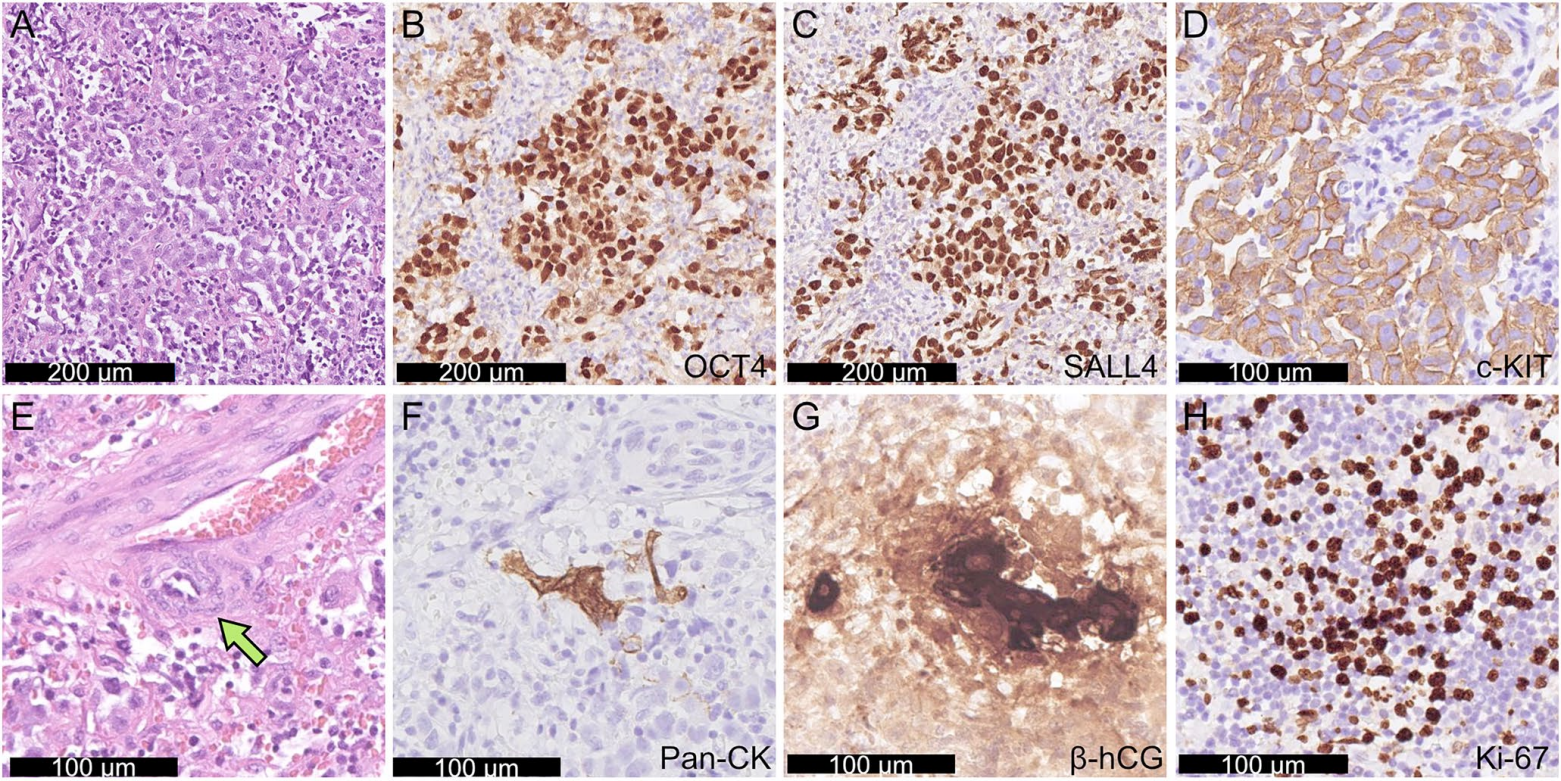
Histology and immunohistochemistry of the metachronous pineal germinoma. (A) H&E shows sheets of large round cells with clear cytoplasm and prominent nucleoli admixed with dense lymphocytic inflammation. (B) OCT4 and (C) SALL4 show diffuse strong nuclear staining. (D) c‐KIT highlights membranous staining in tumor cells. (E) H&E shows an enlarged eosinophilic cell consistent with a syncytiotrophoblastic giant cell (arrow). (F) Pan‐CK highlights focal trophoblastic cells. (G) β‐hCG highlights focal β‐hCG‐positive syncytiotrophoblastic cells. (H) Ki‐67 immunohistochemistry shows a high proliferative index. Scale bars are indicated in each panel.

Pathologic examination of the second pineal mass showed sheets of large round cells with clear cytoplasm and conspicuous nucleoli, admixed with dense lymphocytic inflammation (Figure 4A). Immunohistochemistry showed strong nuclear OCT4 and SALL4 and membranous c‐KIT (Figure 4B–D), while CD30, AFP, and glypican‐3 were negative. Focal enlarged eosinophilic cells consistent with syncytiotrophoblastic giant cells were identified on H&E and were highlighted by pan‐cytokeratin and β‐hCG immunohistochemistry (Figure 4E–G). There were no areas showing a biphasic proliferation of cytotrophoblastic cells and syncytiotrophoblastic giant cells, extensive hemorrhage, or geographic necrosis to suggest a choriocarcinoma component. Ki‐67 showed a high proliferative index (Figure 4H). The findings support a diagnosis of germinoma with focal syncytiotrophoblastic giant cells, with no NGGCT component identified. All examined lesional tissue from the second resection showed germinoma with focal syncytiotrophoblastic giant cells. No significant stromal fibrosis, histologic evidence of postoperative scarring, or residual mature teratoma was identified in the examined sections.

## 4 Discussion

This case adds to reports of metachronous intracranial GCTs after gross‐total resection of a pineal mature teratoma. The second tumor arose in the same site after an asymptomatic interval of 11 years and showed classic germinoma with focal syncytiotrophoblastic giant cells and high proliferative activity. Serum β‐hCG was modestly elevated and AFP was normal, which is typical for germinomas with syncytiotrophoblastic elements. This clinicopathologic pattern reflects β‐hCG production by trophoblastic cells within an otherwise typical germinoma [1].

Metachronous intracranial GCT after resection of a mature teratoma is uncommon but has been documented in 12 patients in reports published from 2001 onward, as summarized in Table 1 [8, 9, 10, 11, 12, 13, 14, 15, 16, 17, 18, 19]. The initial mature teratomas arose in the pineal gland (*n* = 10), third ventricle (*n* = 1), or suprasellar region (*n* = 1), and the interval to the first diagnosis of the metachronous tumor ranged from 6 months to 22 years. The metachronous tumors developed at the original site or elsewhere. Reported sites included the pineal region [10, 11, 15], third ventricle [8, 12, 13, 15], suprasellar or intrasellar regions [8, 15, 16, 17, 18], bilateral basal ganglia [9], and lateral ventricular or subependymal regions [14, 15, 16, 19]. Multifocal ventricular or spinal involvement was also reported [10, 14, 16]. Among the 11 histologically confirmed first metachronous tumors, eight were germinomas, two were immature teratomas, and one was a yolk sac tumor. The remaining patient was treated for a presumed disseminated β‐hCG‐secreting GCT based on imaging and tumor‐marker findings, without histologic confirmation [14]. Nine patients received chemotherapy and radiotherapy, two received radiotherapy alone, and the remaining patient underwent total resection of an immature teratoma; adjuvant treatment was not reported. Outcomes were reported for 11 patients. Ten were alive without reported tumor progression at last follow‐up. The patient with the yolk sac tumor died of progressive disease 15 months after presentation with the metachronous tumor [13]. Among the eight cases with a stated follow‐up duration, follow‐up ranged from 12 months to 10 years.

**TABLE 1:** Reported metachronous intracranial germ cell tumors after resection of a primary intracranial mature teratoma.

References	Age/sex	Initial site	Interval, years	Metachronous tumor/site	Treatment	Outcome/follow‐up
Hirano et al. [8]	8/M	Pineal gland	10	Immature teratoma, neurohypophysis/suprasellar region extending into the third ventricle	Total resection	Follow‐up NR
Kim et al. [12]	26/M	Third ventricle	2.5	Germinoma, third ventricle	Subtotal resection + focal RT	NED 20 months after treatment
Sugimoto et al. [9]	10/M	Pineal gland	3	Germinoma, bilateral basal ganglia	Bx/partial resection + CTx + RT	Radiographic CR after the second CTx cycle; follow‐up duration NR
Utsuki et al. [13]	9/M	Pineal gland	7	Yolk sac tumor, third ventricle	Resection + CTx + RT	Died of tumor progression 15 months after his second hospitalization
Janzarik et al. [10]	9/M	Pineal gland	22	Germinoma, pineal region with ventricular and spinal involvement	Bx + RT	CR, disease‐free 2 years posttreatment
Cardellicchio et al. [14]	14/M	Pineal gland	3	Presumed disseminated β‐hCG‐secreting GCT, ventricular and subependymal lesions	CTx + protocol‐based RT	NED at 35 months after starting therapy
Sakakura et al. [11]	5/M	Pineal gland	11	Germinoma, pineal region	Neuroendoscopic biopsy + CTx + WVRT	CR, no recurrence at 12‐month follow‐up
El Meouchy et al. [15]	6/M	Pineal gland	9	Germinoma, suprasellar extending to ventricles, later pineal region	Bx + CTx + CSI with pineal boost	Marked tumor shrinkage; stable for 10 years on hormone replacement
Han et al. [16]	14/M	Pineal gland	4	Immature teratoma, suprasellar region with ventricular dissemination; later spinal and intraventricular germinomas	Multimodal surgery, CTx, RT, and high‐dose CTx with ASCT	CR; no recurrence (follow‐up duration NR)
Hallenberger et al. [17]	9/M	Pineal region	3.5	Germinoma, intrasellar region/infundibular stalk	Transsphenoidal Bx + CTx + proton RT	No recurrence 16 months after Bx
Kang et al. [18]	7/F	Suprasellar region	0.5	Germinoma, suprasellar region	Surgery + CTx + proton RT	CR; follow‐up duration NR
Dowaki et al. [19]	8/M	Pineal gland	4.6	Germinoma, anterior right lateral ventricular wall	Bx + CTx + whole‐brain RT	Alive without further recurrence 72.8 months after recurrence

*Note:* The table includes cases reported from 2001 onward. A metachronous tumor is a subsequent intracranial GCT arising after resection of a primary intracranial mature teratoma, at the same or a different site. Age refers to the initial diagnosis; interval refers to the first metachronous tumor.

Abbreviations: ASCT, autologous stem cell transplantation; Bx, biopsy; CTx, chemotherapy; CR, complete remission; CSI, craniospinal irradiation; F, female; GCT, germ cell tumor; M, male; NED, no evidence of disease; NR, not reported; RT, radiotherapy; WVRT, whole ventricular RT.

Long‐term surveillance is needed even after gross‐total resection of a mature teratoma. Among 25 patients with recurrent CNS germinoma, 9 (36%) experienced their first recurrence at or beyond 60 months, and the latest recurrence occurred at 230 months [20]. Similarly, Takami et al. reported that 28.6% of germinoma recurrences occurred more than 10 years after treatment [21]. Metachronous germinomas have arisen at the original pineal site more than 10 years after teratoma resection [10, 11] and at suprasellar or intrasellar sites after shorter intervals [15, 17, 18]. Current guidelines do not support routine prophylactic irradiation or chemotherapy after complete resection of mature teratoma solely to prevent later metachronous malignancy, particularly given the risk of long‐term treatment‐related complications in pediatric and young adult patients [3, 21]. In our opinion, a reasonable approach is brain MRI at decreasing frequency for at least a decade, with clinical review for neuroendocrine symptoms that may herald suprasellar disease. Tumor markers can be obtained when symptoms or imaging change, noting that germinomas with syncytiotrophoblastic giant cells may show only modest β‐hCG elevation. In our case, MRIs obtained annually through age 24 years showed no recurrence. After this surveillance period, the patient received care outside our health system, and no further surveillance MRI was obtained during the asymptomatic interval. This highlights practical barriers to long‐term monitoring in young adults after apparently curative resection of a benign lesion. Therefore, counseling patients about the rare risk of late metachronous malignancy may support long‐term surveillance.

This case also illustrates an important diagnostic distinction: focal syncytiotrophoblastic giant cells within germinoma should not be equated with choriocarcinoma. In the present tumor, focal enlarged eosinophilic cells consistent with syncytiotrophoblastic giant cells were highlighted by pan‐cytokeratin and β‐hCG immunohistochemistry, explaining the modest serum β‐hCG elevation. However, the tumor lacked the biphasic proliferation of cytotrophoblastic cells and syncytiotrophoblastic giant cells, extensive hemorrhage, and necrosis expected for choriocarcinoma. In addition, AFP and glypican‐3 were negative, arguing against a yolk sac tumor component, and CD30 was negative, arguing against embryonal carcinoma. These findings support a diagnosis of germinoma with focal syncytiotrophoblastic giant cells [2, 22].

This distinction is clinically important because germinoma with focal syncytiotrophoblastic giant cells is treated as germinoma when no non‐germinomatous component is identified, whereas true mixed GCT or choriocarcinoma would alter risk stratification and treatment intensity. Thus, modest β‐hCG elevation and focal β‐hCG‐positive syncytiotrophoblastic giant cells should be interpreted in the context of morphology and the full immunohistochemical profile [3, 23].

Several mechanisms may explain metachronous germinoma after a teratoma. One possibility is that an occult germ cell marker‐positive population was already present in the initial tumor and later gave rise to the germinoma. In the present case, retrospective review after diagnosis of the metachronous germinoma identified rare atypical cells within or adjacent to the ciliated respiratory‐type epithelium. OCT4 showed multifocal, patchy immunoreactivity in scattered cells along several segments of the epithelial lining. SALL4, c‐KIT, and PLAP showed focal immunoreactivity in corresponding epithelial regions. These findings raise the possibility of an occult germ cell population in the initial tumor, but its biologic relationship to the later germinoma remains uncertain. A similar mechanism has been suggested by Mano et al. who described a germinoma 21 years after resection of an immature pineal teratoma, in which re‐examination of archival slides and cerebrospinal fluid cytology suggested early dissemination and prolonged dormancy [24]. However, no discrete expansile germinoma component was identified in the examined sections in our case, and the biologic relationship between these marker‐positive cells and the later germinoma cannot be proven without paired molecular studies. An additional limitation is the retrospective nature of the immunohistochemical evaluation of the initial tumor. The marker‐positive population was recognized with knowledge of the later germinoma, which may have influenced the interpretation of these cells. Serum AFP and β‐hCG levels before the initial surgery were also unavailable in the medical record, although postoperative values during early follow‐up were within the reference ranges used at those time points. Therefore, these findings raise the possibility of a link to the later germinoma but do not establish a definitive diagnosis of mixed GCT.

A second possibility is multicentric or de novo origin. Primordial germ cells or related precursor cells may remain at more than one midline site and give rise to separate tumors, although the histogenesis of intracranial GCTs remains incompletely resolved [2]. Synchronous pineal and suprasellar disease has been described in large clinical series, supporting the concept that multiple midline sites may be involved [25]. Delayed germinomas after resection of a first lesion have been reported at a different midline site, including neurohypophyseal and basal ganglia locations, and at the original tumor site [7, 9, 10, 11, 12]. In this view, the later pineal germinoma in our patient could represent a separate tumor arising from a quiescent germ cell focus. Recent observations of primordial germ cell‐like cells in the fetal brain provide developmental support for this possibility, but the evidence remains limited [26]. Without paired molecular studies of the initial teratoma and the later germinoma, multicentric or de novo origin cannot be proven.

A third, less common mechanism is malignant transformation. Teratomas can develop somatic‐type malignancies in the tumor bed. This phenomenon is well documented in testicular GCTs [27]. In the intracranial setting, a mature teratoma that later presented as a yolk sac tumor has been reported and interpreted as late malignant change [13]. Germinoma after teratoma is biologically different from somatic‐type transformation, but the clinical message is similar: a benign teratoma does not exclude later malignant disease.

Emerging molecular data provide additional understanding of the biology of intracranial germinomas. Genome‐wide DNA methylation profiling shows that pure germinomas have global low methylation with LINE‐1 hypomethylation, closely resembling migrating primordial germ cells, which supports a primordial germ cell origin [28]. KIT or RAS pathway mutations are frequent in germinomas and are mutually exclusive in large series, indicating early MAPK pathway activation in this lineage [29]. These molecular features alone do not distinguish occult residual neoplasm from multicentric origin. In mixed GCTs, microdissected components can share identical MAPK/PI3K pathway mutations, suggesting divergence from a common ancestor, yet direct proof of clonal relatedness between a resected teratoma and a later germinoma is rarely achievable because diagnostic tissue is limited and the second tumor often arises after many years [28]. In our case, methylation profiling and targeted sequencing of the index teratoma and the later germinoma were not available; therefore, whether the two tumors shared a common clonal origin remains uncertain and represents an important limitation of this report. Paired molecular studies of the index teratoma and the later germinoma, including methylome profiling and targeted sequencing, would be required to test clonal relationships, define timing, and clarify whether dormancy or multifocal origin is more common, and such data could refine surveillance strategies and identify risk markers at the first surgery.

In conclusion, an adult developed a pineal germinoma with focal syncytiotrophoblastic giant cells 11 years after gross‐total resection of a mature teratoma. Retrospective evaluation of the initial tumor identified rare atypical cells on H&E and multifocal, patchy OCT4 immunoreactivity in scattered cells along the respiratory‐type epithelial lining. These findings raised the possibility of an occult germ cell population, although no discrete germinoma component or definitive mixed GCT was identified. This case supports prolonged clinical and MRI follow‐up after resection of a mature teratoma. It also shows that modest β‐hCG elevation and focal β‐hCG‐positive syncytiotrophoblastic giant cells should be interpreted in the context of morphology and the full immunohistochemical profile.

## Ethics Statement

This case report was prepared in accordance with institutional policies and the Declaration of Helsinki. Institutional Review Board approval was not required for a single‐patient case report.

## Consent

Written informed consent for publication was obtained from the patient.

## Conflicts of Interest

The authors declare no conflicts of interest.

## Data Availability

The data that support the findings of this study are available from the corresponding author upon reasonable request.
